# Traumatic injury to vascular prosthetic anastomosis: A case report and diagnostic approach

**DOI:** 10.1016/j.ijscr.2024.110239

**Published:** 2024-09-05

**Authors:** Nulvin Djebbara-Bozo, Emilie Arntoft Pedersen, Maria Arvad Serifi, Christian Nikolaj Petersen

**Affiliations:** a Department of Plastic and Breast Surgery, Aalborg University Hospital, Hobrovej 18-22, 9000 Aalborg, Denmark; b Department of Radiology and Intervention, Kolding Hospital, Sygehusvej 24, 6000 Kolding, Denmark; c Department of Vascular surgery, Aalborg University Hospital, Hobrovej 18-22, 9000 Aalborg, Denmark

**Keywords:** Pseudoaneurysm, Traumatic, Vascular, Prosthesis, Case report

## Abstract

**Introduction:**

Traumatic injury of a vascular prosthesis-to-prosthesis anastomosis leading to an extravasation and pseudoaneurysm is rare. If not identified the complications associated with this condition can lead to high morbidity and mortality and require surgical treatment.

**Case:**

We describe a patient who presented with a tear in prosthesis-prosthesis anastomosis eight years after implantation resulting in a pseudoaneurysm. The patient had a severe fall prior to the non-symptomatic leakage. The complication was successfully treated by re-lining the graft with a new anastomosis at the Department of Vascular and Endovascular Surgery, Kolding Hospital in Denmark.

**Discussion:**

Cause of tear is speculated to be due to weakness at site of reconstruction, fabric degradation, and/or degradation of suture material.

**Conclusion:**

Late prosthesis-prosthesis anastomosis tear caused by a traumatic event is rare. In the event of a late tear, anamnesis and histological analysis of involved material is important.

## Highlights

• Traumatic injury to a prosthesis-to-prosthesis anastomosis can lead to a leak and pseudoaneurysm formation.
• Leakage can be asymptomatic, making it potentially lethal to limb and life.
• UL and CT are considered gold standard for the diagnosis of a pseudoaneurysm.

## 1 Introduction

Pseudoaneurysms, also labeled false aneurysms, are localized disruptions in relation to an artery, continuously leaking arterial blood into an adjacent cavity. This differs from a true aneurysm that contains all the layers of the arterial wall [1]. The cause of pseudoaneurysms differs and can be iatrogenic by penetrating arterial access for endovascular procedures or sometimes non-iatrogenic due to trauma or bacterial infection [2,3].

Morbidity associated with a pseudoaneurysm located to prosthesis site is linked to a range of potential complications, including ischemia, infection, and organ failure. Ischemic events may occur due to reduced blood flow to vital organs. The mortality associated with this condition is also significant. Untreated, the pseudoaneurysm may continue to grow, increasing the risk of sudden rupture, which can lead to massive bleeding and death.

To reduce the morbidity and mortality associated with pseudoaneurysm, early diagnosis and prompt treatment are crucial. This may involve the use of advanced imaging techniques such as ultrasound (US) and computed tomography scanning (CT) to identify and assess the pseudoaneurysm, as well as surgical intervention to repair or replace the damaged prosthesis. Additionally, it is important to monitor the patient closely postoperatively to detect and manage any complications promptly. Anastomotic pseudoaneurysm formation in the suture line between a native artery and prosthetic graft is described in the literature and is a known complication in reconstructive vascular surgery with the majority in the femoral region. Complications from pseudoaneurysms include venous thrombosis, infection, formation of AV-fistula and rupture. These require treatment typically by open surgery with arterial reconstruction, although endovascular therapy is occasionally an option [4].

Only a few cases of pseudoaneurysm in prosthesis-prosthesis anastomosis are mentioned where none have been described outside of the aorta, to our knowledge [5,6]. In this report, we describe how a pseudoaneurysm forms in the anastomosis site between an axillofemoral bypass and fem-fem bypass after a blunt blow to the anastomosis site caused by a traumatic fall.

The research subject presented in this article has provided informed and written consent to publish personal information and illustrations.

## 2 Report

An 88-year-old woman with a medical history of polymyalgia rheumatica, hypertension, atherosclerosis and hypercholesterolemia, was referred to the vascular surgery department by her general practitioner due to a bulge in her right groin region. The bulge had developed and gradually increased in size after a severe fall that affected the right side of her body, from shoulder to ankle, seven months prior to her visit at the department.

The patient had a long history of vascular procedures having undergone four vascular surgeries from 1981 to 2015. In 1981, an aortobiiliac bypass was placed due to intermittent claudication. By February 2005, a CT showed bilateral femoral pseudoaneurysms in the common iliaca arteries at the site of the anastomosis that resulted in a new surgery connecting a Gorex 8 prosthesis end-to-end from the legs of the old aortobiiliac bypass to the common femoral artery bilaterally. By 2007, she presented with rectal bleeding where a CT revealed an aorto-enteric fistula. The old aortobiilic bypass was removed and a bilateral axillofemoral bypass with Dacron material was implanted connecting the distal prosthesis site end-to-side with the old iliac-femoral prosthesis from 2005, bilaterally. There were no signs of infection in this distal part of the two iliac-femoral prosthesis. The right groin wound was complicated with infection managed with conservative wound treatment and peroral antibiotics.

In 2015, the patient complained about pain in her left leg during rest. Clinically, peripheral pulse was not palpable and a CT-scan showed that the left axillofemoral bypass was occluded. She underwent her fourth surgery, connecting a polytetrafluoroethylene (PTFE) crossing bypass from the distal part of the right axillofemoral bypass to the left deep femoral artery. The postoperative period was without complications.

Upon her arrival to the department, she was received by a doctor from the department of vascular surgery. Clinically, a 7 × 5 cm round, hard, non-pulsatile bulge was observed in her right groin. The ancle-brachial index (ABI) bilaterally was 50–60 mmHg, and she did not experience leg pain at rest. Due to inactivity, her walking distance was limited, but she did not report any ischemic symptoms while walking. There were no systemic symptoms of bleeding. A bedside US revealed extra-vascular fluid communicating with the prosthesis. She was subsequently referred to radiology for further evaluation.

An experienced interventional radiologist, conducted a US scan initially using Doppler-flow and B-mode. The US revealed an open right axillofemoral bypass with an inhomogeneous surrounding mass (7 ⨯ 5.3 cm), consistent with a pseudoaneurysm. No Doppler-flow was detected within the pseudoaneurysm except for a punctate area.

**Fig. 1:**
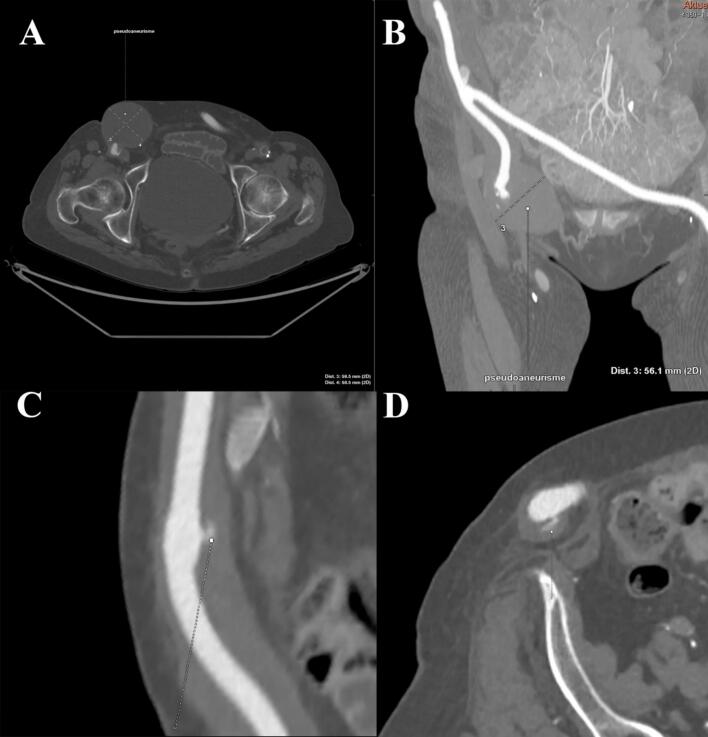
A and B: CT-angiography in axial and coronal plane, with Multiplanar Reconstruction (MPR) showing pseudoaneurysm forming in the anastomosis site between an axillofemoral bypass and fem-fem bypass. C and D: CT-angiography in arterial phase (axial plane), showing contrast extravasation from the defect of the posterior part of the prosthesis, causing the pseudoaneurysm.

To further clarify the findings, a CT-angiography was performed (see Fig. 1). The protocol was performed in early, arterial, venous, and late venous phase (diagnostic delay of 13, 60, and 90 s, respectively). The venous phase is important for detecting small and/or slow leaks. The scan confirmed the findings from the US, showing a 16 mm mass around the prosthesis in the right groin with slight density gain of 45 HU on arterial and venous phase. Additionally, a 6 × 9 cm accumulation with a density of 38 HU in empty phase, which indicative of a hematoma/pseudoaneurysm, was observed more distally.

**Fig. 2:**
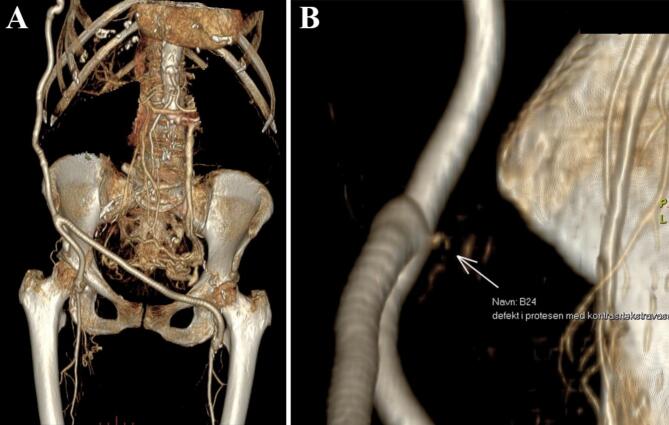
A and B: 3D reconstruction in coronal and axial. B clarifies the defect in the prosthesis with contrast extraversion.

A 3D image was constructed to further visualize the defect and contrast extraversion (see Fig. 2). Contrast leakage was noted from a small defect in the posterior wall of the prosthesis just before its bifurcation ventral to the crista iliaca.

**Fig. 3:**
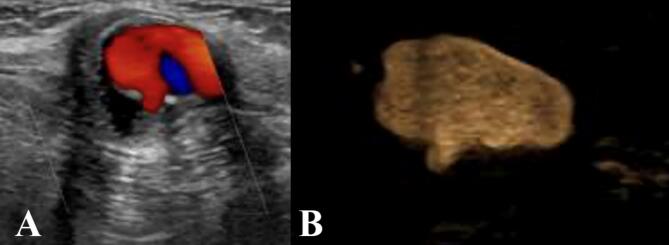
A and B: Respectively show US with Doppler-flow and B-mode images, revealing the defect in the prosthesis. B-flow mode focuses is designed to display the movement of blood as it flows through the vessel in a dynamic manner. It enhances the contrast between blood and surrounding tissue, enabling improved visualization of blood flow patterns and detection of abnormalities.

To confirm and demonstrate the prosthesis defect, a focused US was performed, revealing a 1 mm defect in the posterior wall of the aortobifemoral bypass just before its bifurcation, with a small jet flow in and out of the accumulation surrounding the prosthesis. The pulsatile flow was confirmed using Doppler-flow and B-flow, thereby validating the leak and defect in the prosthesis (see Fig. 3).

Patient was planned for elective surgery 4–5 weeks after her first contact with the department. During surgery, the right end-to-side anastomosis between the axillofemoral bypass and PTFE bypass connected to the contralateral deep femoral artery was visualized. A defect on the posterior side of the anastomosis was found. A new and similar anastomosis was reconstructed by re-lining the grafts and the pseudoaneurysm was removed. Operation and postoperative weeks were without complications. There was no imaging tests conducted after surgery, only clinical follow-up and surveillance. Had the patient presented in a hemodynamically unstable condition, the case would require urgent assessment and stabilization followed by urgent surgical intervention.

## 3 Discussion

The occurrence of a vascular prosthesis-prosthesis-anastomosis tear due to a traumatic fall is rare, with no similar previously documented cases. In this case, anastomosis was hand-sewn between a PTFE graft and Dacron graft using continuous polypropylene suture. Dacron was introduced in vascular surgery in the 1950s and have since gained widely practice as a vascular prosthetic conduit [7]. Along with the PTFE graft, they are the most commonly used allografts in vascular surgery [8]. Although, there have been case reports about graft disruption, it has never been a major problem and the main attention has been preventing clotting or infection of graft material [9]. In this case, the graft material was macroscopically intact, while the suture line in the anastomosis broke. We speculate that this was due to the severe fall the patient endured. When constructing an anastomosis, it is crucial to use appropriate suture material e.g. non-absorbable and suitable thickness to avoid complications.

The patient in this case had undergone three years of surveillance after the last surgery, with no significant findings and therefore properly concluded years prior to this case. There is a lack of evidence to determine optimal follow-up protocol for patients undergoing vascular surgery [10]. Regarding imagining, optimal surveillance after open lower extremity arterial revascularization depend on the type of intervention, whether it is an anatomic or extra-anatomic bypass or with prosthetic or autogenous material. Surveillance generally begins immediately after surgery and continues at 3, 6, and 12 months and then every 6 to 12 months thereafter [11]. Surveillance includes both clinical examination, physiologic testing such as ABI and direct imaging modalities, however Doppler US provides the ability to accurately assess all aspects of an arterial reconstruction over time and is therefore most often applied to bypass graft [12].

Generally prosthetic grafts have been shown to have a durability of around 5–10 years [13,14], but vary significantly based on type of graft used, location of implantation, surgical technique, the patient's comorbidities and underlying vascular conditions. Special risk groups are diabetic patients, patients with a previous vascular history and smokers. Surveillance should be similarly tailored to patient risk profiles.

While the underlying mechanism causing the disintegration of the anastomosis remains unknown, in the following, potential explanations are postulated.

One potential factor is fabric degradation. The surgeons did not report a tear in the prosthesis fabric, but it is conceivable that fabric degradation could have contributed to the leakage. A case report in 2012 detailed the prosthesis fabric tear of a PTFE graft occurring five years post-implantation, resulting in an abdominal aortic aneurysm [15]. However, the specific cause of the fabric's degradation in that instance remained unknown.

Another study documented a case involving the rupture of polypropylene suture at the anastomosis site, occurring five years following the implantation of a fabric vascular graft. Upon examination, electron microscopy revealed that the suture had a tapering-off breakage point, due to the suture being stretched out gradually and finally fractured [16]. Regrettably, in this instance, the sutures were not subjected to histopathological examination, leaving us only to speculate whether a similar mechanism occurred in our patient's case. It is plausible that the sutures in our patient underwent gradual stretching, potentially exacerbated by the force of the severe fall, ultimately leading to tear.

In this article, we illustrate the utilization of US and CT-angiography in diagnosing the pseudoaneurysm. These imaging techniques are considered highly reliable for viewing vascular abnormalities, including pseudoaneurysms. US allows for real-time visualization of blood vessels and can identify even small pseudoaneurysms with high accuracy, while CT-angiography allows detailed imagining of blood vessels in three dimensions displaying precise location of the pseudoaneurysm. Combining US and CT therefore provides a comprehensive and reliable approach to the diagnosis of pseudoaneurysms and is considered the gold standard [17,18].

This report underlines the critical importance of conducting a thorough anamnesis when assessing a patient. In cases of a late tear at the anastomosis site, it is essential to histologically examine the prosthesis and suture material. Additionally, it is beneficial to consider the potential consequences of combining two types of different prosthetic materials, and whether this could create a weak area at the anastomosis site. A comprehensive understanding and analysis of factors leading to a late anastomosis tear is crucial in all situations, as it can inspire further research and ultimately lead to improve patient outcomes.

## Disclaimer

Consent was obtained from the patient to publish this case report including information, pictures, and radiological images. No competing interest to be declared. No conflict of interest to be reported. We received no financial support in publishing this case or in any steps. No AI function was used.

The case report was reported in line with SCARE checklist [19].

## Ethical approval

Ethical approval was not required for this study.

## Funding

No funding.

## Guarantor

Nulvin Bozo.

## CRediT authorship contribution statement

Study design: Nulvin Bozo
Introduction, writing: Nulvin Bozo
Case report, writing: Nulvin Bozo
Case report, writing: Emilie Arntoft
Pictures: Emilie Arntoft + Maria Serifi
Discussion, writing 70%: Nulvin Bozo
Discussion, writing 30%: Emilie Arntoft
Revision and supervision: Christian Pedersen.

## Declaration of competing interest

No conflict of interest.
